# Knockdown of P3H4 inhibits proliferation and invasion of bladder cancer

**DOI:** 10.18632/aging.102732

**Published:** 2020-02-04

**Authors:** Lin Hao, Kun Pang, Hui Pang, Junjie Zhang, Zhiguo Zhang, Houguang He, Rongsheng Zhou, Zhenduo Shi, Conghui Han

**Affiliations:** 1 Department of Urology, Xuzhou Central Hospital, Xuzhou 221009, Jiangsu, China; 2 The Third Affiliated Hospital of Soochow University, Changzhou 213000, Jiangsu, China; 3 Central Laboratory, Xuzhou Central Hospital, Xuzhou 221009, Jiangsu, China; 4 College of Science and Technology, Jiangsu Normal University, Xuzhou 221009, Jiangsu, China

**Keywords:** P3H4, bladder cancer, ETV4, transcription factor

## Abstract

The prolyl 3-hydroxylase family member 4 (P3H4) (alias SC65) is implicated in a variety of physiological and pathological processes. However, little is known about the role of P3H4 in tumors. This study aimed to investigate the role of P3H4 in bladder cancer (BC) and the regulatory mechanisms that influence its expression. P3H4 was highly expressed in BC tissues. Knockdown of P3H4 inhibited BC cell proliferation, cell cycle, migration and invasion *in vitro*, and inhibited BC growth *in vivo*. We also found that ETV4 bound directly to the promoter region of P3H4 and activated its transcription. Furthermore, overexpression of ETV4 rescued the inhibition of proliferation and invasion induced by PH4 silencing. ETV4 was significantly overexpressed in the BC tissues. In conclusion, P3H4 functioned an oncogene role in BC progression, and ETV4 bound directly to the P3H4 promoter region to regulate its transcription.

## INTRODUCTION

Bladder cancer (BC) is the most common malignancy of the genitourinary system, with more than 430,000 new patients worldwide affected each year [1, 2]. BC is also the seventh leading cause of cancer-related deaths worldwide, accounting for 2.8% of these deaths [3]. Surgical resection combined with radiotherapy or chemotherapy has been applied to BC treatment [4]. However, patients receiving aggressive treatment still face the status quo of easy recurrence, poor prognosis, low survival rate and poor quality of life [5, 6]. Therefore, finding new and effective therapeutic targets is urgent and important.

The prolyl 3-hydroxylase family member 4 (P3H4) (alias SC65) is originally identified as a protein associated with the association complex (SC) [7]. However, no further evidence supports this point. Subsequent studies detect antibodies against P3H4 in cases of interstitial cystitis [8], membranous nephropathy [9], prostate cancer [10], and meningeal cancer [11]. P3H4 has also been identified as an autoantigen associated with these diseases. Furthermore, distinct functions of P3H4 are reported separately. For example, P3H4 acts as an endoplasmic reticulum (ER) protein that regulates bone mass homeostasis and skin fragility [12]. Studies have shown that P3H4 is expressed at high levels in bone, cartilage and skin. It also forms a stable complex with P3H3 in ER and interacts with lysyl hydroxylase 1 and CYPB [13, 14]. Additionally, P3H4 is an adaptor protein that links myelin protein zero (P0) and the activated C kinase 1 (RACK1) receptor [15], a gene that is up-regulated in the hippocampus during sleep [16], and is possibly a PTEN-interacting protein [17]. In general, P3H4 is involved in a variety of physiological and pathological processes. However, little is known about the role of P3H4 in BC.

In the present study, we found that P3H4 was significantly overexpressed in BC tissues, and knockdown of P3H4 inhibits BC cell proliferation, cell cycle, migration and invasion. ETV4 regulated P3H4 transcription by binding directly to its promoter region and was involved in the regulation of BC progression. Overexpression of ETV4 rescued the inhibition of proliferation and invasion induced by PH4 silencing.

## RESULTS

### P3H4 is highly expressed in BC

**Figure 1:**
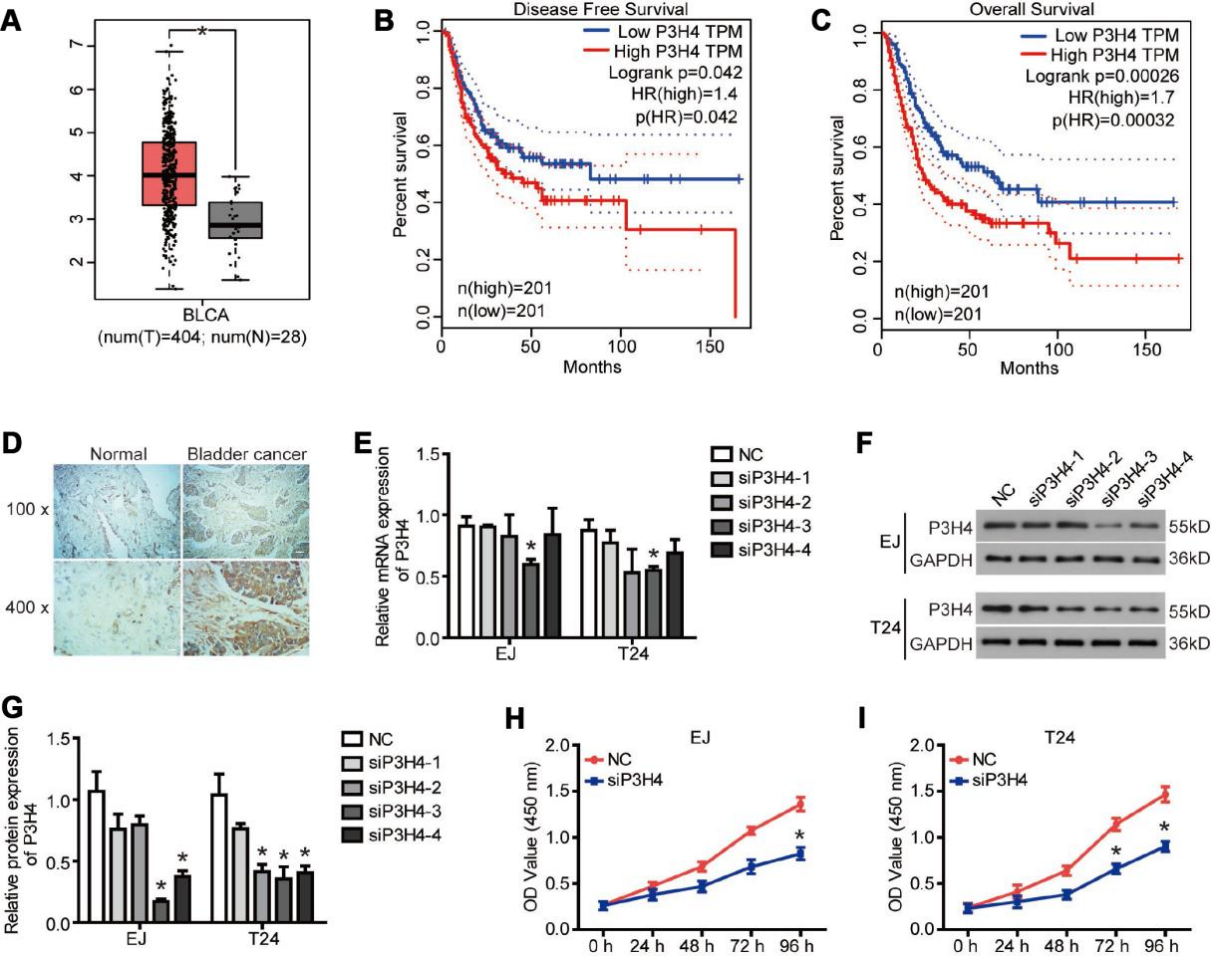
**P3H4 is highly expressed in BC.** (**A**)The boxplot of P3H4 mRNA level. Red and gray boxes represent bladder urothelial carcinoma (BLCA) tissue and normal bladder tissue, respectively. The data came from the GEPIA database. Disease free survival (**B**) and overall survival (**C**) percentage of BLCA patients with high or low P3H4 expression. (**D**) P3H4 expression in bladder cacner (BC) tissues and adjacent normal tissues was examined by Immunohistochemical (IHC) analysis. After siRNA targeting P3H4 (siP3H4) was transfected into EJ and T24 cells, P3H4 mRNA (**E**) and protein (**F** and **G**) expression were detected by RT-qPCR and western blot, cell proliferation (**H** and **I**) were measured by CCK8 assays. *P<0.05.

To reveal the role of P3H4 in BC, we first examined the P3H4 expression in BC tissues by database analysis and IHC analysis. The GEPIA online analysis website (http://gepia.cancer-pku.cn/) was used to analyze P3H4 mRNA levels in 404 patients with bladder urothelial carcinoma (BLCA) and 28 healthy volunteers. As shown in Figure 1A, the mRNA levels of P3H4 were significantly higher in BLCA patients compared to the healthy volunteers. The disease-free survival and overall survival of BLCA patients with low P3H4 expression was obviously higher than those of BLCA patients with high P3H4 expression (Figure 1B and 1C). In addition, we collected 32 BC tissues and 26 normal tissues to measure P3H4 expression. As shown in Figure 1D and Table 1, P3H4 was high expressed in BC tissues compared to normal tissues. Furthermore, statistical analysis demonstrated that high expression of P3H4 in BC tissues was related to gender (Table 2). Moreover, the incidence of BC in males is 3-4 times that of females. These results suggest that P3H4 may play an oncogene function in BC.

**Table 1:** P3H4 expression in BC compared with normal tissue.

**Group**	**n**	**P3H4 expression**		***P***
		**Low (n%)**	**High (n%)**	
BC	32	8 (25.0)	24 (75.0)	0.005^*^
Normal	26	16 (61.5)	10 (38.5)	

**Table 2:** P3H4 expression associated with the clinicopathological parameters in BC.

**clinicopathological parameters**	**n**	**P3H4 expression**		***P***
		**Low (n%)**	**High (n%)**	
Gender				
Male	26	4 (15.4)	22 (84.6)	0.009*
Female	6	4 (66.7)	2 (33.3)	
Age (years)				
<65	11	3 (27.3)	8 (72.7)	0.830
≥65	21	5 (23.8)	16 (76.2)	
Tumor diameter (cm)				
<4	15	3 (20.0)	12 (80.0)	0.539
≥4	17	5 (29.4)	12 (70.6)	
Clinical stages				
1-2	9	2 (22.2)	7 (77.8)	0.820
3-4	23	6 (26.1)	17 (73.9)	

### Knockdown of P3H4 inhibits BC cell proliferation by impeding cell cycle progression

**Figure 2:**
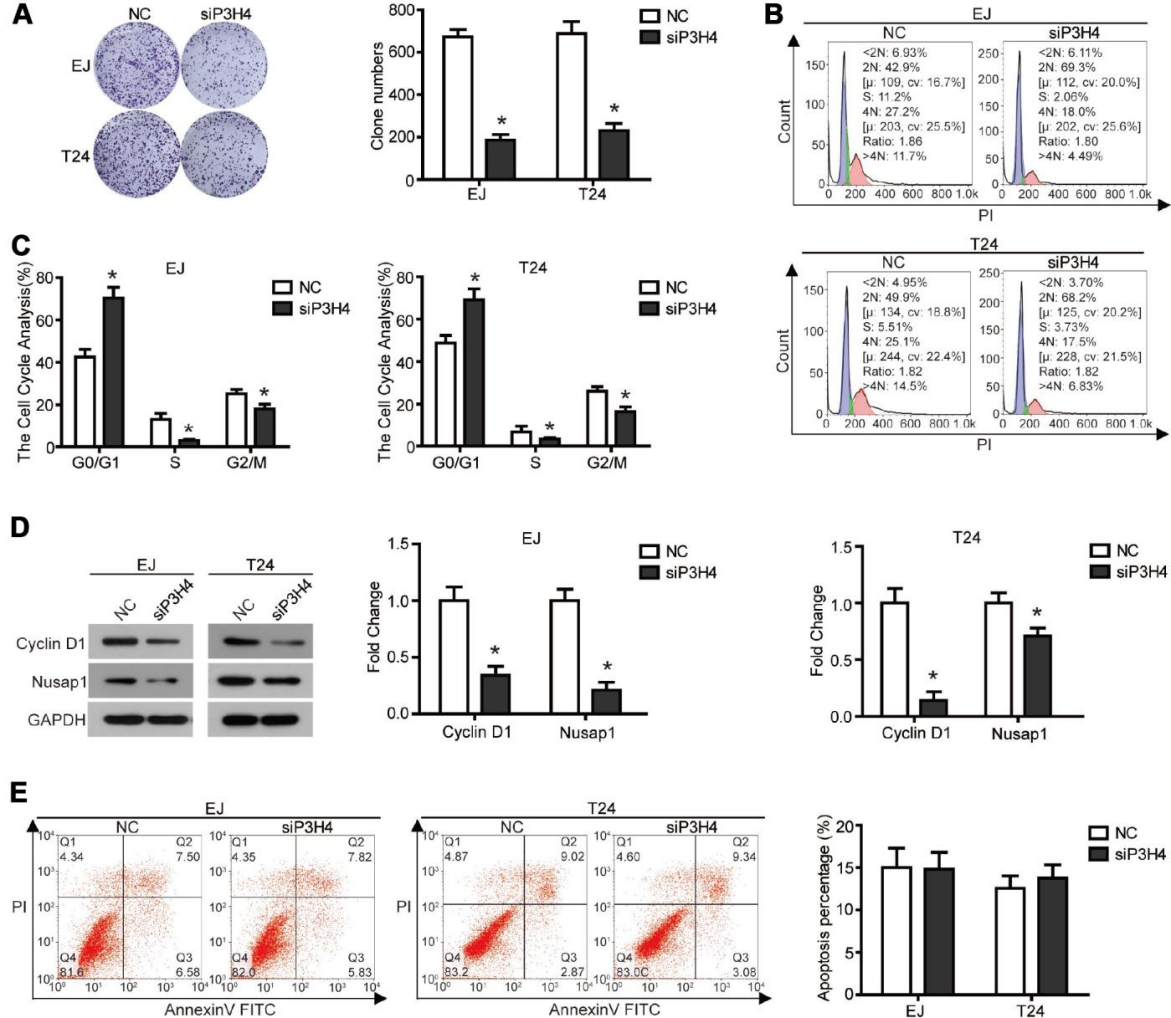
**Knockdown of P3H4 arrested cell cycle in the G1 phase and thus inhibited proliferation, but had no effect on apoptosis.** After siP3H4 was transfected into EJ and T24 cells, (**A**) the clonality was evaluated by clone formation assay, (**B** and **C**) cell cycle was detected by flow cytometry, (**D**) the protein expression of cell cycle-related proteins Cyclin D1, Nusap1 and p70 were measured by western blot, (**E**) apoptosis was examined by flow cytometry. *P<0.05.

To expose the action of P3H4 in BC cells’ biological function, expression of P3H4 was down-regulated with siRNA (Figure 1E, 1F), and siRNA-4 targeting P3H4 (siP3H4) was used in subsequent experiments. As shown in Figure 1H and 1I, CCK8 assay showed that proliferation of EJ and T24 cells was inhibited when siP3H4 was transfected into cells. Similarly, colony formation assay revealed that knockdown of P3H4 inhibited the colony forming ability of EJ and T24 cells (Figure 2A). Furthermore, we detected the action of P3H4 knockdown on the cell cycle and apoptosis of BC cells. As shown in Figure 2B and 2C, the proportion of cells in G0/G1 phase increased while the proportion of cells in S and G2/M phase decreased, when siP3H4 was transfected into cells. These results indicate that knockdown of P3H4 impedes cell cycle in the G0/G1 phase. In addition, transfection of siP3H4 reduced the protein levels of Cyclin D1, Nusap1 and p70 (Figure 2D). Cyclin D1 is a key protein that regulates the G1 phase of the cell cycle [18]. Nusap1 is an indispensable microtubule and chromatin binding protein, which regulates the dynamics of kinetochore microtubules, governs chromosome oscillations, and ensures the normal progress of cell cycle [19, 20]. Finally, flow cytometry detection found that knockdown of P3H4 had no effect on the apoptosis of BC cells (Figure 2E). These results demonstrate that the knockdown of P3H4 can inhibit BC cell proliferation by arresting cell cycle progression, but that it has no effect on the apoptosis of BC cells.

### Knockdown of P3H4 inhibits BC cell migration and invasion by affecting EMT progression

**Figure 3:**
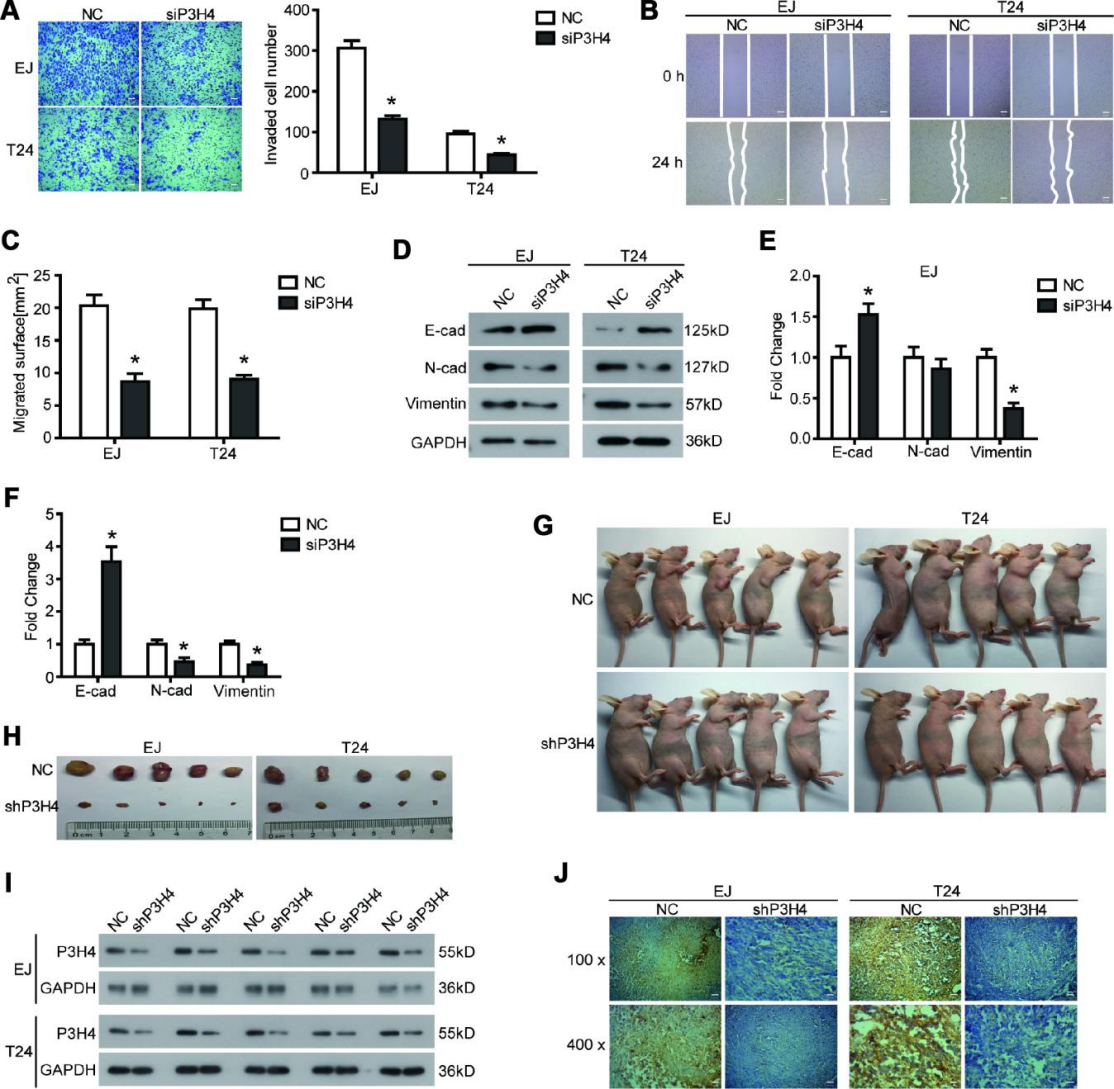
**Knockdown of P3H4 inhibits BC cell migration and invasion in vitro and BC growth in vivo.** After siP3H4 was transfected into EJ and T24 cells, (**A**) the invasion ability was determined by Transwell assay, the migration ability was examined (**B**) and quantified (**C**) by wound healing assay, (**D**–**F**) the expression of EMT-related proteins was detected by western blot. (**G**) Images of xenograft nude mice after 4 weeks of treatment. (**H**) Images of subcutaneous tumor. The expression of P3H4 in subcutaneous tumors was examined by western blot (**I**) and IHC (**J**). *P < 0.05.

In addition to the effects on BC cell proliferation, we also examined the effects of P3H4 knockdown on BC cell motility. Transwell assay showed that compared to NC, fewer EJ and T24 cells could pass through the matrigel and invade the lower surface of the chamber membrane (Figure 3A). Additionally, wound healing assay revealed that transfection of siP3H4 caused wound healing of EJ and T24 cells to be slower (Figure 3B and 3C). Moreover, the knockdown of P3H4 reduced protein levels of N-cadherin and Vimentin, as well as increased the protein levels of E-cadherin (Figure 3D–3F). Decreased E-cadherin expression, increased N-cadherin and increased Vimentin were the most important sign change of the Epithelial-Mesenchymal transition (EMT) [21]. EMT plays a pivotal role in the primary infiltration and secondary metastasis of a variety of solid tumors [22].

### Knockdown of P3H4 inhibits BC growth in vivo

In addition to cells in vitro, a subcutaneous xenograft model was used to further assess the biological role of P3H4 in BC progression. As shown in Figure 3G, 3H, knockdown of P3H4 in EJ and T24 cells significantly inhibited BC growth. Western blot and IHC analysis also demonstrated that the expression of P3H4 was down-regulated by the shP3H4 transfection (Figure 3I, 3J).

### ETV4 is the transcription factor of P3H4

**Figure 4:**
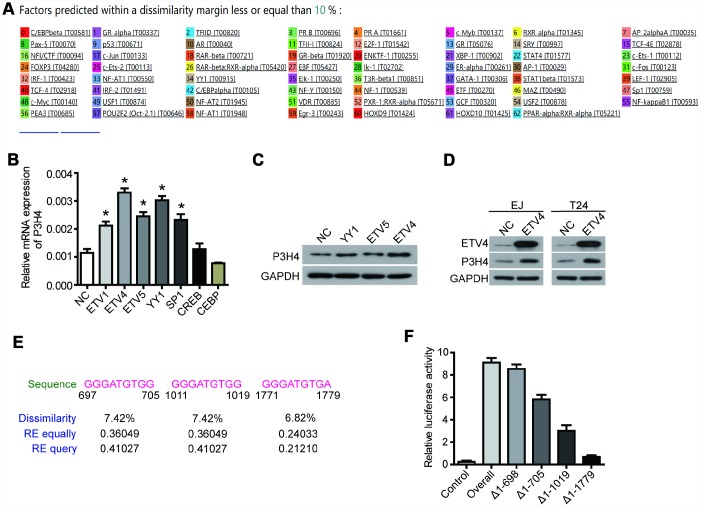
**ETV4 is the transcription factor of P3H4.** (**A**) The PROMO database predicted a variety of P3H4 transcription factors. After transfected with transcription factor overexpression plasmid respectively, P3H4 mRNA and protein expression in EJ cells were detected by RT-qPCR (**B**) and western blot (**C**). (**D**) After transfected with ETV4-overexpression plasmid, the expression of ETV4 and P3H4 was examined by western blot. (**E**) Three predicted ETV4 binding P3H4 promoter sites. (**F**) The site where ETV4 bound directly to the P3H4 promoter region was measured by luciferase activity assay. *P < 0.05.

The PROMO database predicted a variety of P3H4 transcription factors (Figure 4A), of which ETV1, ETV4, ETV5, YY1, SP1, CREB, and CEBP were selected for subsequent testing. Their overexpression plasmids were transfected into EJ cells, respectively. RT-qRCP detection revealed that overexpression of the transcription factors ETV4, ETV5, and YY1 significantly enhanced the mRNA level of P3H4 (Figure 4B). Western blot results also showed that ETV4 could better enhance the expression of P3H4 (Figure 4C, 4D). Furthermore, the promoter analysis revealed the presence of three binding regions for ETV4 on the promoter of P3H4, located at 697-705, 111-1019, and 1771-1779, respectively (Figure 4E). Fragment deletion plasmid Δ1-698 (1-698 bp deletion), Δ1-705 (1-705 bp deletion), Δ1-1019 (1-1019 bp deletion), and Δ1-1779 (1-1779 bp deletion) were constructed and co-transfected into 293T cells with ETV4-overexpression plasmid. Fluorescein assay results indicated that ETV4 was able to bind to three predicted sites (Figure 4F).

### ETV4 is essential for P3H4 to affect BC cell proliferation and motility.

**Figure 5:**
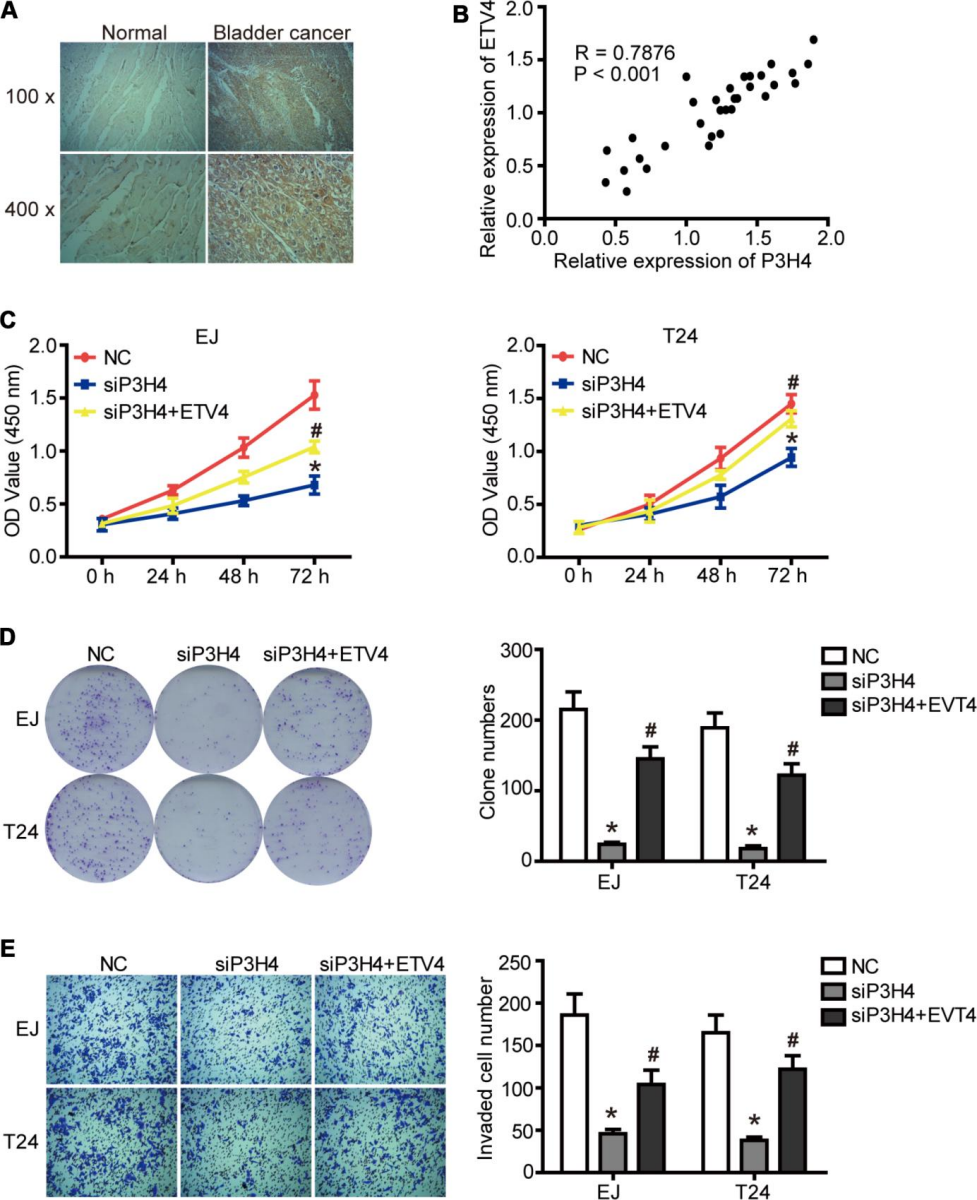
**ETV4 is essential for P3H4 to affect BC cell proliferation and motility.** (**A**) ETV4 expression in BC tissues and adjacent normal tissues was examined by IHC. (**B**) The correlation analysis of P3H4 and ETV4 expression. After siP3H4 and ETV4-overexpression plasmids were co-transfected into EJ and T24 cells, cell proliferation were measured by CCK8 assays (**C**) and clone formation assay (**D**), invasion ability was determined by Transwell assay (**E**). * *P*<0.05.

Given that ETV4 may be an important transcription factor for P3H4, we further examined the expression of ETV4 in BC tissues. As shown in Figure 5A and Tables 3, 4, ETV4 was significantly high expressed in BC tissues compared to normal tissues, and ETV4 expression was related to clinical stage of BC. Furthermore, there was a significant positive correlation between the expression of P3H4 and ETV4 (Figure 5B, P < 0.001, R = 0.7876). In addition, ETV4 overexpression partially restored the inhibitory of siP3H4 transfection on proliferation, colony formation and invasion ability of EJ and T24 cells (Figure 5C–5E).

**Table 3:** ETV4 expression in BC compared with normal tissue

**Group**	**n**	**ETV4 expression**		**P**
		**Low (n%)**	**High (n%)**	
BC	33	12 (36.4)	21 (63.6)	0.001^**^
Normal	27	22 (81.5)	5 (18.5)	

**Table 4:** ETV4 expression associated with the clinicopathological parameters in BC

**Clinicopathological parameters**	**n**	**ETV4 expression**		**P**
		**Low (n%)**	**High (n%)**	
Gender				
Male	27	9 (33.3)	18 (66.7)	0.765
Female	6	3 (50.0)	3 (50.0)	
Age (years)				
≤65	15	5 (33.3)	10 (66.7)	0.974
<65	18	7 (38.9)	11 (61.1)	
Tumor diameter (cm)				
≤4	20	6 (30.0)	14 (70.0)	0.773
<4	12	5 (41.7)	7 (58.3)	
Clinical staging				
1-2	9	7 (77.8)	2 (22.2)	0.009^*^
3-4	24	5 (20.8)	19 (79.2)	

## DISCUSSION

In the present study, we discovered the role of P3H4 in BC, and found that ETV4 can act as a transcription factor to influence BC progression by regulating P3H4 transcription.

Database analysis and IHC results showed abnormally high expression of P3H4 mRNA and proteins in BC tissues. Li et al also detected P3H4 mRNA expressions in 44 pairs of BC tissues and adjacent normal tissues by qRT-PCR, as well as discovered that P3H4 mRNA were highly expressed in BC tissues [23]. Their findings are consistent with our results.

Knockdown of P3H4 arrested the EJ and T24 cell cycle in the G1 phase, thus inhibiting cell proliferation. However, this no effect on cell apoptosis. In addition, knockdown of P3H4 also impeded migration and invasion of both EJ and T24 cells by inhibiting the EMT process. The xenograft nude mouse model also showed that knockdown of P3H4 inhibited the growth of subcutaneous BC tumors. These results indicate that P3H4 may play an oncogene function in BC progression. Although studies have displayed that P3H4 may be associated with several tumors (prostate cancer [10] and meningeal cancer [11]), this study was the first to conduct experiments in vivo and in vitro to determine the role of P3H4 in BC.

Furthermore, ETV4 could regulated the P3H4 transcription in EJ and T24 cells. ETV4 (ETS Variant 4) is a transcription factor belonging to the EST family and is specifically involved in the carcinogenesis of various tissues, including pancreatic cancer, breast cancer and intestinal cancer [24–26]. However, little is known about the role of ETV4 in BC. ETV4 is frequently up-regulated in tumors and acts as an oncogenic factor via different mechanisms. IHC analysis also found a significant up-regulation of ETV4 expression in BC. By fusion with other genes, ETV4 regulates genome transcriptional pattern and carcinogenesis [27]. For example, TMPRSS2-ETV4, KLK2-ETV4 and CANTT-ETV4 [27, 28]. ETV4-related gene fusion may be an early event in prostate cancer and could define a new molecular subtype of prostate cancer [28]. On the other hand, ETV4 can directly bind to the promoter region of target genes and regulate their transcriptional activity. In pancreatic cancer and breast cancer, ETV4 directly upregulates the transcription of cyclin proteins, such as cyclin D1 [26] and cyclin D3 [29] to promote cell proliferation. MMPs, such as MMP13 and urokinase plasminogen activator (uPA) are also transcriptionally regulated by ETV4, which are involved in the ETV4-driven malignant phenotypes in prostate cancer [30] and breast cancer [25]. In the present study, our results of western blot and Luciferase activity assay demonstrated that ETV4 can directly bind to the promoter of P3H4 and activate transcription of P3H4. Moreover, three binding sites of the ETV4 and P3H4 promoter regions were found. Overexpression of ETV4 rescued the inhibition of proliferation and invasion induced by PH4 silencing. These results indicate that ETV4 can participate in the regulation of BC progression by transcriptionally regulating the expression of P3H4.

In conclusion, P3H4 regulated proliferation, cell cycle, migration and invasion, playing an oncogene role in BC progression. In BC cells, ETV4 bound directly to the P3H4 promoter region to regulate its transcription.

## MATERIALS AND METHODS

### Tissues specimens

32 fresh BC tissues and 26 normal adjacent tissues were obtained from BC patients who underwent a urethral cystectomy (TURB) at Xuzhou Central Hospital in 2018. Normal tissues were excised from 5 to 7 cm away from the tumor. The specimens were immediately frozen in liquid nitrogen and stored at -80 °C. The pathological samples were reassessed by an experienced urinary tract division (ALB) to confirm the diagnosis. The hospital ethics committee approved the study and all patients signed written informed consent.

### Immunohistochemistry (IHC)

Collected clinical samples were immersed in formalin for 24 h at 4 °C, followed by paraffin embedding. Paraffin blocks were cut into 4 μm sections by a cryo-cutting machine and IHC was performed to evaluate P3H4 or ETV4 protein expression. IHC was performed using non-biotinylated rabbit polyclonal anti-human P3H4 (_Proteintech_, Manchester, UK, 15288-1-AP, 1:100) and rabbit polyclonal anti-human ETV4 (Proteintech, Manchester, UK, 10684-1-AP, 1:200). Van-Clear (Hongci., Shanghai, China) and concentration gradient ethanol were used for the dewaxing of paraffin sections. Antigen retrieval was performed by microwave pretreatment in 0.01 M citrate buffer for 10 min. After being blocked with 5% sheep serum at room temperature for 1 h, the sections were incubated with the primary antibody, and incubated with Enzyme-labeled goat anti-mouse/rabbit IgG polymer (160101405L, Maixin., Shanghai, China). The immune response was visualized by a Reinforced DAB Chromogenic Kit (1705252031, Maixin., Shanghai, China), and hematoxylin was used for counterstaining. Finally, images were obtained using an upright microscope system (Nikon, JAPAN).

The total P3H4 or ETV4 immunostaining score was calculated by multiplying the positive staining cell ratio ® and staining intensity score (S) which were given by two pathologists blinded to the clinical parameters. R was divided into four grades: 0 (<5%, negative), 1 (5-25%, sporadic), 2 (25-50%, focal), 3 (> 51%, diffuse). S was also divided into four grades: 0 (negative), 1 (weak), 2 (moderate), 3 (strong). Finally, a total of 0-3 was considered to be low staining, while 4-9 was considered to be high staining.

### Cell culture

BC cell lines EJ and T24, as well as 293T cells were obtained from the Cell Bank of the Chinese Academy of Sciences (Shanghai, China). All cells were cultured in DMEM medium (Gibco, NY, USA) containing 10% fetal bovine serum (FBS; Gibco, Australia) and 1% penicillin–streptomycin with 5% CO_2_ at 37 °C in a humidified incubator.

### Plasmid, construction of the lentivirus and transfection

Four small interfering RNA (siRNA) sequences (Ruibo, Guangzhou, China) were synthesized to target P3H4 mRNA. Their sequence was as follows: siRNA-1: GGGCUGUGAAGCUCUACAATT, siRNA-2: CCAA GUAUCUCAACUACUATT, siRNA-3: CGGCCAUA GCAGAUCUCUUTT, siRNA-4: GGAGCCUGAGGA UGCCCUATT.

SiRNA targeting P3H4 sequence was transformed into short hairpin RNA (shRNA), which was cloned into a pLVX-shRNA2 green fluorescent protein (GFP) vector. The recombinant plasmid was transfected into the 293T cells together with two lentiviral packaging plasmids to generate lentivirus. After incubation for 18 h and 42 h, the lentivirus from the culture medium was collected and concentrated by Lentiviral Packaging Kits (TR30037, Origene, Billerica, MA, USA). For cell infection, EJ and T24 cells at a concentration of 5 × 10^4^/well in a six-well plate were incubated with shRNA-P3H4 lentivirus (P3H4 knockdown group) or negative control lentivirus (NC), with a multiplicity of infection (MOI) of 50, lasting 24 h. Pools of stable transductions were generated by selection using puromycin (4 μg/ml) for 2 weeks. Transduction efficiency was determined using fluorescence microscopy (MicroPublisher 3.3 RTV, Olympus, Tokyo, Japan), western blot and qRT-PCR analysis of GFP expression.

The ETV1, ETV4, ETV5, YY1, SP1, CREB and CEBP cDNA sequences were cloned into the pcDNA3.1 vector, respectively, and were transfected into EJ and T24 cells to up-regulate the expression of target gene. The P3H4 promoter sequence 697-1779 (1-698 bp deletion), 706-1779 (1-705 bp deletion), and 1019-1779 (1-1019 bp deletion) were cloned into the pGL3.0 vector, respectively, and were transfected into EJ and T24 cells together with pcDNA3.1-ETV4 plasmid. Transfection of all siRNA and plasmids was performed by Lipofectamine 2000 (Invitrogen, USA).

### qRT-PCR

Total mRNA from cells was extracted using a TRizol reagent (Invitrogen, Carlsbad, CA, USA), and reverse transcribed to cDNAs by the PrimeScript Reverse Transcription Reagent Kit (TaKaRa, Dalian, China). P3H4 mRNA expression was quantitatively analyzed using SYBR Premix Ex Taq™ (TaKaRa, Dalian, China) with the 2^−ΔΔCt^ method. β-actin as an internal reference. The primers used were as follows: P3H4 forward: 5′- CGGGCTGTGAAGCTCTACAA-3′, P3H4 reverse: 5′- GCGTCAGATAGGGCATCCTC-3′; β-actin forward: 5′- CCCGAGCCGTGTTTCCT-3′, β-actin reverse: 5′- GTCCCAGTTGGTGACGATGC-3’. ′

### Western blot

Total proteins from cells and tissues were dissolved with RIPA buffer and quantified with the BCA Protein Assay Kit (Tiangen, Beijing, China). 15 μg of proteins were separated through 10% SDS-PAGE gel and then transferred onto PVDF membranes. After being incubated with 5% fat-free skim milk for 1 h at room temperature, PVDF membranes were incubated with primary antibodies at 4 °C overnight and subsequently incubated with secondary antibody at room temperature for 2 h. Immunoreactive bands were developed using Enhanced chemiluminescence kit (Amersham, Little Chalfont, UK).

Anti-P3H4 (1:1000, 15288-1-AP), anti-E-cadherin (1:1000, 29874-1-AP) and anti-ETV4 (1:1000, 10684-1-AP) were purchased from Proteintech (Manchester, UK, USA). Anti-Cyclin D1 (1:1000, ab16663), anti-Nusap1 (1:1000, ab169083), anti-p70 (1:1000, ab131526), anti-N-cadherin (1:1000, ab18203), anti-Vimentin (1:1000, ab8978), anti-GAPDH (1:1000, ab181602) were purchased from Abcam (Cambridge, MA, USA).

### CCK8 assay

5×10^3^ cells were seeded in each well of a 96-well plate and cultured overnight. After 0 h, 24 h, 48 h and 72 h of transfection, 100 μl of fresh medium containing 10 μl CCK8 solution (Beyotime Biotechnology, Shanghai, China) was added to each well and incubated at 37 °C for 2 h. The OD value at a wavelength of 450 nm was measured with a microplate reader.

### Colony forming assay

200 transfected cells were normally cultured in each well of a six-well plate. After two weeks, cells were fixed with methanol and stained with 0.1% crystal violet. Visible colonies were counted and pictured.

### Flow cytometry detection for cell cycle

After 48 h of transfection, cells were trypsinized (without EDTA), and resuspended in cold PBS. Subsequently, cells were fixed in cold 70% ethanol at -20 °C for 24 h. Fixed cells were incubated with RNase A for 30 min, and stained with propidium oxide for another 30 min in the dark by a Cell Cycle Analysis Kit (Beyotime Biotechnology, Beijing, China). Cell cycle progression was immediately detected using a flow cytometer, and analyzed using FCS Express 4 software (De NovoSoftware, Los Angeles, CA).

### Flow cytometry detection for cell apoptosis

After 48 h of transfection, cells were trypsinized and collected by the Annexin V binding buffer. Subsequently, cells were double stained with V-FITC and propidium iodide (BD Biosciences, Franklin Lakes, NJ, USA), then measured by BD FACS Canto II (BD Biosciences). Data was analyzed using FlowJo software.

### Transwell assay

Transwell chambers (BD Biosciences) were used to evaluate the invasion ability of EJ and T24 cells. The upper surface of chambers was coated with Matrigel. 200 μl of cell suspension with a concentration of 1x10^6^ cells/ml was added into the upper chamber. The lower chambers were filled with 600 μl of DMEM containing 10% FBS. After incubation at 37 °C for 24 h, cells on the upper chambers were removed, and invaded cells on the lower surface of the chambers were fixed and stained with 0.1% crystal violet. Invaded cells were counted and photographed under a microscope (200x magnification, Nikon TE2000).

### Wound healing assay

Wound healing assay was performed to measure the migration ability of GC cells. Cells were grown to confluence in 6-well plates. A sterile plastic tip was used to create wounds. After washing with PBS, cells were cultured in serum-free medium for 48 h. Images were taken using a microscope. The average of five random widths of each wound was measured for quantification.

### Xenografts in mice

All experimental procedures were approved by the Ethics Committee. Approximately 1x10^7^ cells were injected subcutaneously into the armpits of female athymic BALB/C nude mice (4-6 weeks old, 18-22 g, 5 mice per group). Tumor growth was monitored weekly. After 4 weeks, the mice were euthanized and photographed. The subcutaneous tumor mass removed, and a picture was taken. A portion of the tumor mass was fixed and embedded for IHC, and a portion of the tumor mass was used for western blot.

### Luciferase activity assay

The pGL3.0 recombinant plasmid and pcDNA3.1-ETV4 plasmid were co-transfected into EJ and T24 cells using Lipofectamine 2000. After 48 h, cells were harvested and luciferase activity was detected using a luciferase assay system (Promega, Madison, Wisconsin, USA).

### Statistical analysis

Statistical analysis was performed as the mean ± SD and conducted using SPSS 22.0 (SPSS, IBM, Beijing, China) and GraphPad Prism 6 (GraphPad, San Diego, CA). Differences were calculated with the Student's t-test between two groups or with one-way ANOVA among multiple groups. *P* < 0.05 was considered statistically significant.
